# Obesity, Adipose Tissue, and Inflammation Answered in Questions

**DOI:** 10.1155/2022/2252516

**Published:** 2022-01-22

**Authors:** Vanessa A. Guerreiro, Davide Carvalho, Paula Freitas

**Affiliations:** ^1^Department of Endocrinology Diabetes and Metabolism, Centro Hospitalar e Universitário de São João, Porto 4200-319, Portugal; ^2^Faculty of Medicine, Universidade do Porto, Porto 4200-319, Portugal; ^3^Instituto de Investigação e Inovação em Saúde, Universidade do Porto, Porto 4200-135, Portugal

## Abstract

*Background*. Obesity is a global health problem of epidemic proportions, which is characterized by increased adipose tissue (AT) mass and significant repercussions in different body apparati and systems. AT is a special connective tissue, which contains several types of cells, in addition to adipocytes, and is a highly active endocrine and immune organ, which directly modulates many processes, including energy balance, metabolism, and inflammation. *Summary*. In this paper, the authors list and attempt to answer in a brief and simple way several questions regarding the complex relationships between obesity, adipose tissue, and inflammation, with the objective to provide an easy way to understand the main changes that occur in this pathological state. The questions are the following: Is adipose tissue only made up of adipocytes? Are adipocytes just a reservoir of free fatty acids? Do different types of fatty tissue exist? If so, which types? Can we further subcategorize the types of adipose tissue? Is it possible to form new adipocytes during adulthood? What is the role of inflammation? What is the role of macrophages? Are macrophages central mediators of obesity-induced adipose tissue inflammation and insulin resistance? What causes macrophage infiltration into adipose tissue? What is the role of hypoxia in AT alterations? Is there cross talk between adipocytes and immune cells? What other changes occur in AT in obesity? Does metabolically healthy obesity really exist? Is this a benign condition? *Key messages*. Obesity is a complex disease with numerous metabolic consequences, which are mainly the result of dysfunction that occurs in the adipose tissue of patients with this pathology. Understanding the pathophysiology of AT and the changes that occur in obesity would contribute to a better approach to patients with obesity, with the inherent medical implications that could result from this.

## 1. Introduction

During the past few decades, the prevalence of both obesity (which is defined in Caucasians to be a body mass index (BMI) of over 30 kg/m^2^) and being overweight (25 ≤ BMI < 30 kg/m^2^) has been increasing rapidly in Western societies and in developing countries [1] and is a health problem of epidemic proportions.

Since 1980, the prevalence of obesity has doubled in more than 70 countries. Over 600 million adults were obese in 2015, with high BMI accounting for approximately 4 million deaths worldwide [2].

Obesity is a chronic, complex, and heterogeneous disease, which remains a global health concern across the globe, with an associated increased risk of multiple conditions [3, 4]. Depending on the degree and duration of weight gain, obesity can progressively cause and/or exacerbate a wide spectrum of comorbidities. Obesity is one of the key factors for the development of metabolic diseases, such as type 2 diabetes *mellitus* (T2DM), hyperlipidemia, hypertension, and atherosclerosis, as well as cancer and other chronic diseases—including liver dysfunction, subfertility, respiratory, musculoskeletal, and psychosocial disorders [5].

The risk of developing these problems rises exponentially when BMI is over 30 kg/m^2^, which is associated with an increase in the relative risk of premature death—primarily from cardiovascular disease (CVD). In individuals with BMI of between 25 and 29.9 kg/m^2^ (overweight), the risk of premature mortality appears to be mainly influenced by fat distribution. Indeed, whereas intra-abdominal fat accumulation (android obesity) is associated with a higher risk of cardiometabolic diseases, independent of the level of BMI, fat accumulation in the subcutaneous regions of hips, thighs, and lower trunk (gynoid obesity) is less harmful [6, 7]. Furthermore, some [2, 8], but not all [9], of the studies suggest that being overweight could be associated with lower all-cause mortality than normal weight (BMI 18.5 to 25 kg/m^2^). This is known as the obesity paradox, which suggests that additional factors, possibly metabolic ones, can affect the risk of death within BMI categories.

## 2. Is Adipose Tissue Only Made Up of Adipocytes?

No. AT represents a special connective tissue that contains adipocytes (which comprise 35–70% of AT mass [10]) and several other cell types, such as preadipocytes, nerve tissue, fibroblasts, endothelial cells, and various immune cells surrounded by capillary and innervation networks, which function together as an integrated unit. Immune cells within adipose tissue are largely categorized into innate (macrophages, neutrophils, eosinophils, and mast cells) or adaptive (CD4, CD8, natural killer T cells—NKT—and B cells) immune cells [11, 12].

## 3. Are Adipocytes Just a Reservoir of Free Fatty Acids?

No—on the contrary to what was previously thought. Although lipid storage and both thermal and mechanical insulations are all classical functions of AT, the traditional view of AT as a passive reservoir for energy storage has been progressively replaced by the identification of AT as a multifunctional, essential, complex, and highly active metabolic, immune, and endocrine organ [10, 13], which directly modulates many processes, including energy balance, metabolism (for example: AT is the largest storage organ for energy in the form of triacylglycerol), inflammation, and bone metabolism [14, 15].

The endocrine functions of adipose fall into two broad categories: (i) to secrete proteins (for example: cytokines and cytokine-related proteins, other immune-related proteins, proteins involved in the fibrinolytic system, proteins for lipid metabolism or transport, and leptin that controls appetite), which have many different metabolic effects, as can be seen below; and (ii) the production of enzymes involved in the metabolism of steroid hormones (for example: cytochrome P450-dependent aromatase, 17*β*-hydroxysteroid dehydrogenase, and 11*β*-hydroxysteroid dehydrogenase type 1) [13]. This pleiotropic ability is mainly due to the capacity of adipocytes to not only respond to afferent signals from the central nervous system (for example: TSH receptor and GH receptor) and traditional hormone systems (for example: insulin receptor and glucagon receptor) but also express and secrete various bioactive peptides (hormones and cytokines), which are known as adipocytokines (cytokines that are produced by AT) or adipokines (a broader term that includes many other factors) [10], which act at both the local (paracrine/autocrine) and systemic (endocrine) levels, with many different endocrine functions. Indeed, AT secrete a large number of adipokines at the same time as expressing receptors for most of these substances, which enables an extensive crosstalk in response to specific external stimuli or metabolic alterations [10]. The important function of AT is reinforced by the unfavorable metabolic consequences of both AT excess and deficiency [16].

Several adipokines are produced in adipose tissue, including the following: (i) leptin, which suppresses appetite, increases insulin sensitivity, and promotes lipolysis [17]; (ii) adiponectin, which also increases insulin sensitivity, suppresses hepatic glucose output, and has anti-inflammatory and antifibrotic actions [18]; (iii) resistin, which participates in proinflammatory response and is involved in the development of insulin resistance [19]; vaspin (visceral adipose tissue-derived serpin; serpin A12); cytokines, such as IL-6—which promote insulin resistance, the recruitment of macrophages in AT, local, and systemic inflammation [20], and tumor necrosis factor-*α* (TNF-*α*)—which induces insulin resistance in AT through the alteration of the normal insulin signaling pathway. The list of these substances is continually growing and numerous new markers of AT dysfunction are now known to exist in addition to the traditional adipokines, which are classically associated with AT inflammation, such as IL-8 [21], WISP1 [22], apelin [23], chemerin [24], and DPP4 [25]—which increase insulin resistance; angiopoietin 2 [26]; and omenti-1 [27]—which increase insulin sensitivity. Adipocyte cell size correlates positively with the secretion of proinflammatory adipocytokines [14] and also induces a dysregulated production of these bioactive substances with the accumulation of adipocytes, which in turn contributes to the development of a metabolic syndrome [14].

## 4. Do  Different Types of Fatty Tissue Exist? If So, Which Types?

Yes, white, brown, beige, and pink adipocytes exist [28]. Adipocytes were initially classified into two main types, according to their morphology, location, and function, namely: white and brown adipocytes [29]. Nowadays, the current classification includes a third category of adipocytes—which are called beige or brite (brown-in-white) adipocytes [30]. Furthermore, during pregnancy, lactation, and post-lactation, subcutaneous white adipocytes convert to milk-producing glands formed by lipid-rich elements that can be defined as pink adipocytes [31].

White adipocytes are usually large, round cells, which contain a big unilocular lipid droplet surrounded by a thin layer of cytoplasm with few mitochondria [10]. Their main functions are lipolysis, energy storage in the form of fat, and adipokine secretion. These cells can be found on subcutaneous, visceral, retroperitoneal, inguinal, or gonadal tissues [32]. White adipose tissue (WAT) is the major adipose organ in mammals and represents 10% or more of the body weight of healthy adult humans [33]. The physical location of WAT determines its metabolic identity and core functions, as can be seen below.

Brown adipocytes are polygonal cells with multilocular lipid droplets in the cytoplasm, as well as a large quantity of mitochondria [31]. Their main biological function is thermogenesis, although they can also store energy in the form of fat and can secrete adipokines, albeit to a lesser extent than white adipocytes [34]. The mitochondria of brown adipocytes contain a unique mitochondrial protein, which induces heat production by uncoupling respiration from ATP synthesis—the uncoupling protein 1 (UCP1) [31]. In newborns, this type of fat is abundant in the interscapular region and neck, with the function to prevent hypothermia. There is an involution of this tissue during childhood, and it is believed that only vestigial amounts of this tissue exist in adulthood [35]; however, some studies have revealed that adults have active brown fat in some regions, such as neck, mediastinum, supraclavicular, paravertebral, and suprarenal fat [32, 36].

Brown adipocytes are derived from mesenchymal stem cells, which express a myogenic factor 5 (Myf5), whereas white adipocytes are predominantly derived from mesenchymal stem cells, which do not express this transcription factor [37]. Myf5 is a gene, which is expressed during embryonic myogenesis, which is one of the core transcriptional factors involved in muscle development. Myf5+-expressing cells differentiate into myocytes or brown adipocytes, whereas, on the other hand, cells without this factor differentiate into pericytes or into white adipocytes. Beige fat cells originate from endothelial and perivascular cells within WAT and have a unique gene signature [38], which is different from that present in both the other types of fat cells [32, 39]. Beige adipocytes express low levels of UCP1 under basal condition, although they demonstrate thermogenic properties when subject to *β*-adrenergic stimulation after exercise or cold exposure, which produces high amounts of this protein [30].

The recent use of single-cell RNA sequencing (scRNAseq) has proved to be a helpful methodology to detect cellular heterogeneity, which is a promising tool for understanding the development and plasticity of adipose tissue [40] enabling the identification of further subcategories of adipocytes through the adoption of this technique [41].

## 5. Can We Further Subcategorize the Types of Adipose Tissue?

Yes, it is possible to categorize adipose tissues in subcutaneous adipose tissue (SCAT) and visceral adipose tissue (VAT).

Obesity is a heterogeneous condition, with a large level of variability in fat deposition and the associated comorbidities. In humans, WAT is located in many distinct depots and it is beneficial to subcategorize this AT into two broad categories, namely: subcutaneous (beneath the skin, which is predominant in gluteofemoral depots) and visceral (which accounts for up to 10–20% of total fat in men, and 5–8% in women, and is predominant in the abdominal cavity) AT [42].

Visceral obesity, which can be determined by measures such as increased waist circumference (WC) or waist-to-hip ratio, as well as elevated intra-abdominal fat area in cross-sectional abdominal imaging, is strongly associated with increased cardiometabolic risk [43], although, on the other hand, the expansion of subcutaneous fat only acts as a minor contributor or can even, in some cases, decrease the risk of metabolic dysfunction [44]. Indeed, the correlation between waist circumference and BMI is not direct and it has been demonstrated that a stronger association between metabolic and cardiovascular disorders exists with WC than with BMI [45].

Although AT volume is undoubtedly linked to cardiovascular risk, it has been shown that differences in the quality of fat tissue are closely linked with this risk, independent of total fat volume [45]. In addition, it has been proved that a regional variation in WAT distribution is a better predictor of metabolic and cardiovascular risk than overall adiposity [46].

Anatomical and physiological differences between these two types of adipose tissue both help to explain the increased metabolic and cardiovascular risks associated with abdominal obesity. The type of adipocytes, and their lipolytic activity, endocrine function, and response to hormones all differ between subcutaneous, visceral depots, and bone marrow. Visceral depots are rich in inflammatory cells [47] when compared with SCAT—and they represent a buffer for excess energy intake and act as a metabolic protector where the excess of free fatty acids (FFAs) and glycerol are stored as triglycerides [48]. When the storage capacity of the SCAT is exceeded, or if its ability to generate new adipocytes is impaired, either due to stress (such as high cortisol levels—cortisol activates lipoprotein lipase, which in turn triggers lipid accumulation in adipocytes through highly dispersed receptors, mostly found in intra-abdominal visceral depots) or a genetic predisposition (where some genetic variants are associated with a more or less favourable adiposity, such as genes involved in lipid metabolism), an accumulation of fat is found in visceral fat tissue or in other areas outside the SCAT [49], such as muscle, liver, and bone, which are not adapted to lipid storage, causing toxic effects—a process known as ectopic lipid accumulation [50]. Furthermore, while VAT drains directly to the liver—through the portal vein—due to its anatomical position, venous drainage of the SCAT occurs through systemic veins. Accordingly, the portal drainage of visceral fat provides direct hepatic access to FFAs and adipokines secreted by visceral adipocytes, which in turn activates hepatic immune mechanisms and leads to the production of inflammatory mediators, such as C-reactive protein [51]. VAT contains a substantial number of large adipocytes in contrast to SCAT. As adipocytes grow, they become dysfunctional and more insulin-resistant [52]. Insulin resistance, which occurs when cells in muscles, fat, and liver, does not respond well to insulin and cannot easily absorb glucose from blood—which could be one of the most important factors that links VAT to cardiovascular risk (for example, insulin resistance leads to impaired fatty acid metabolism, which results in increased triglyceride content and VLDL secretion from liver, where this excess of lipids accumulates in various organs, such as in the cardiomyocytes—where they are shunted into nonoxidative pathways with subsequent lipotoxicity, which leads to the alteration of cellular signaling and cardiac structure. In addition, insulin resistance also has a deleterious effect on vasculature).

Furthermore, adipocytes also have molecular differences, such as a high density of glucocorticoid [53] and androgen receptors [48] in VAT. VAT is also more sensitive to catecholamine-induced lipolysis, is less sensitive to *α*2-adrenergic receptor-dependent inhibition of lipolysis [54], and is more metabolically active than SCAT—with higher rates of lipolysis and a higher rate of insulin-stimulated glucose uptake. Small adipocytes in SCAT have a high avidity for the uptake of FFAs and triglycerides, acting as a “buffer,” which can absorb circulating FFAs and triglycerides during the postprandial period [55].

Differences also exist between both adipose tissue depots with regard to the capacity of synthesis and release adipokines. While SCAT is the major source of leptin [56], adiponectin is expressed more in VAT [48]. Bone marrow fat contributes to a greater insulin sensitivity in premenopausal women, possibly due to increased adiponectin secretion [57]. VAT has more inflammatory cells and therefore generates proinflammatory cytokines more easily [47]. Angiotensinogen (a peptide hormone that causes vasoconstriction) and the plasminogen activator inhibitor (PAI)-1 (the main inhibitor of the fibrinolytic system) content are higher in VAT than in SCAT [58].

## 6. Is It Possible to Form New Adipocytes during Adulthood?

Yes, adipose tissue growth is a tightly regulated biological process, where mass is determined by both hypertrophy (increase in the size of existing adipocytes) and hyperplasia (increase in cell number, with the formation of new adipocytes through differentiation of preadipocytes) of cells.

The number of adipocytes is primarily determined early on in life (after birth and during adolescence) and is mostly stable during adult life [59]. Adipocyte turnover is sustained throughout life by maintaining a delicate balance between adipogenesis and apoptosis. Recent studies have demonstrated that visceral and subcutaneous fat depots can enlarge during weight gain, with new adipocytes emerging from the differentiation of preadipocytes (which are fibroblast-like and are located in the perivasculature) under prolonged caloric excess, and that they can contribute to the expansion of AT [60]. Hypertrophy is the main contributor to the growth of adipose tissue in adulthood [61], in order to meet the need to accommodate an excessive fat load in the case of obesity. Hyperplasia is correlated with beneficial metabolic properties, although it contributes less to this necessity for fat accumulation—as it occurs in cells with a low fat storage capacity. The creation of smaller adipocytes is correlated with increased angiogenesis, as well as with reduced areas of hypoxic stress on the adipose depot and subsequent inflammation [62, 63]. On the contrary, hypertrophy is associated with the development of metabolic complications [64] because increased adipocyte volume is associated with a dysfunction in membrane proteins, impairment of mitochondrial function, and the generation of a proinflammatory milieu, with hypoxic areas, and consequent cell death [65].

Apoptosis and autophagy are two forms of programmed cell death in the adipose tissue of obese subjects [66, 67]. On the one hand, apoptosis is characterized by cell shrinkage, chromatin condensation, membrane bleeding, nuclear DNA fragmentation, and apoptotic body formation [66], and on the other hand, autophagy is characterized by sequestering cytosolic organelles and proteins in autophagosomes, which are then translocated to lysosomes for degradation [68]. Obesity is associated with the activation of both pathways of adipocyte death, namely apoptosis and autophagy. Previous data strongly suggest that adipocyte apoptosis constitutes a key initial event—which contributes to obesity-associated AT inflammation and insulin resistance [69]. On the contrary, autophagy is essential for normal adipogenesis (due to its participation in controlling intracellular homeostasis, and also due to its contribution to regulating innate and adaptive immune responses), and it has been found that an increased level of autophagy in the adipose tissue of obese patients can attempt to limit the excessive adipocyte hypertrophy and inflammation, in order to prevent adipose tissue dysfunction [70].

Adipose tissue in obese individuals is characterized by various prominent features which are not typically observed in lean individuals. The pathological expansion of AT in the first situation is associated with local inflammation, fibrosis, and with a more proinflammatory adipokine profile—which ultimately promotes insulin resistance and obesity-associated metabolic decline [71].

## 7. What  Is the Role of Inflammation? What Is the Role of Macrophages? Are They Central Mediators of Obesity-Induced Adipose Tissue Inflammation and Insulin Resistance?

In the case of obesity, it has been previously demonstrated that cytokine production by expanded AT leads to high serum levels of proinflammatory cytokines, which in turn induce the activation of IKK*β*/NF*κ*B and JNK pathways, leading to insulin resistance in adipocytes and hepatocytes [11]. Accordingly, in the cases of obesity, the inflammatory responses of AT can play a crucial role in mediating obesity-induced insulin resistance, with various innate and adaptive immune cells in AT being apparently involved in the regulation of AT inflammation and insulin resistance. Although it has been demonstrated that adipocytes are key regulatory cells for the determination of remodeling (dynamic changes in cellular composition and function) and inflammation of AT through cytokine secretion and antigen presentation to T cells (adipocytes can function as antigen-presenting cells), significant evidence also points toward the crucial role of macrophages in such local changes, as can be seen below [32, 72].

Macrophages are phagocytes, which reside in AT and have a dual role, changing their status to support immune responses, obesity development, and related diseases. They perform various roles, including scavenging cellular debris derived from apoptotic cells, remodeling the extracellular matrix, and regulating angiogenesis, as well as playing a key role in the maintenance of AT homeostasis [73]. In obesity, there is an additional infiltration of monocytes and macrophages into AT. In visceral lean AT of humans, macrophages comprise about 10% of stromal vascular cells, although their proportion increases to about 40% of these cells in the case of obesity [74].

A model of “phenotypic switching” of macrophages in AT of obesity was initially proposed, where there is a transformation in the polarized states of macrophages from an anti-inflammatory type M2 macrophages (induced by Th2 cytokines, such as IL-4, IL-10, and IL-13)—which primarily exist in lean adipose tissue and express high levels of arginase-1 (which inhibit nitric oxide synthase activity) and also secrete anti-inflammatory cytokines (such as IL-10 and IL-1 receptor antagonists) [73, 75]—to a more proinflammatory “classically activated” M1 type (both macrophages residing in AT). In this model, M2-polarized macrophages can help preserve normal adipocyte function by promoting tissue repair and angiogenesis in the increasing AT mass, contrary to the classically activated M1 macrophages (which are induced by lipopolysaccharide (LPS) and Th1 cytokine IFN-*γ*) that dominate in states of overnutrition and express a repertoire of proinflammatory factors, such as TNF-*γ* and IL-6, while demonstrating a positive correlation with insulin resistance [76, 77]. Initially, this model was a useful model; however, advances in the understanding of a spectrum of macrophage activation have challenged its accuracy *in vivo* [78]. It is now evident that obesity-associated macrophages include highly plastic cell populations, whose immunophenotype depends on their stimuli [78]. The exact number and functions of macrophages in obese AT are constantly evolving, and the mechanisms that define their unique activation states are not fully clear. Nevertheless, it is now evident that more than one population of macrophages exists in obese AT and that these macrophages can have surface markers that resemble neither M1 nor M2 macrophages, but rather a state of metabolic activation (MMe) which is induced by diverse metabolic stimuli (e.g., free fatty acids, high insulin, and high glucose) [78, 79]. Changes in the microenvironment and inflammatory state lead to macrophage tissue infiltration and its acquisition of a metabolic activation state (macrophage polarization), through the induction of proteins involved in lipid metabolism, which in turn transform these macrophages in cells that allow them to adequately buffer their environment against the excessive lipids. These recruited macrophages differ in their distribution, transcriptomic programming, and functional characterization when compared with resident macrophages [78]. Proteomic analysis of MM1, MM2, and MMe revealed a totally distinct cell surface marker among these cells, where MMe overexpresses ABCA1, CD36, and PLIN2 proteins, which are involved in lipid metabolism [79].

Infiltration of macrophages into expanding AT could have a pivotal role in the inflammatory response of AT in obesity, and also for induced insulin resistance, whereby macrophages are required to create a permissive environment for the multitude of adverse effects of AT expansion [72, 78]. However, further evidence has demonstrated that AT inflammation could be a consequence of insulin resistance, rather than of its etiology [80].

## 8. What Leads to Macrophage Infiltration into Adipose Tissue? What Is the Role of Hypoxia in AT Alterations?

Several factors can contribute to this recruitment, such as the necrosis of adipocytes, leptin, FFAs, and hypoxia—which could be a key regulator of AT changes.

The exact trigger for the chronic inflammatory state of AT in obesity is not well understood, although several mechanisms have been proposed, which could contribute to macrophage recruitment in AT, such as the necrosis of adipocytes—which is driven by adipocyte hypertrophy and accelerated by obesity—and the subsequent formation of crown-like structures (CLSs), with macrophages surrounding the necrotic adipocytes [62, 72, 81]. Furthermore, there is a lot of evidence supporting the role of chemokines, such as the MCP-1/CCR2 pathways [11]—which are derived from macrophage and/or hypertrophic adipocyte—in promoting macrophage mobilization from bone marrow into tissues [82, 83]. For example, adipocytes produce high levels of TNF-*α*, which in turn stimulates the production of MCP-1 by both preadipocytes and endothelial cells, leading to macrophage recruitment by AT [84]. Leptin is other chemotactic factor (by means of: leptin receptors on migrating cells; the influx of intracellular calcium in macrophages; and the activation of janus kinase/signal transducers and activators of transduction [JAK/STAT] as well as mitogen-activated protein kinase [MAPK] and phosphatidylinositol 3-kinase [PI3K] pathways), which could contribute to this recruitment [85].

Another proposed mechanism involves FFAs—which are released from hypertrophic adipocytes through lipolysis during fasting. Some of these FFAs are stored in lipid droplets in the liver, while others can, in situations of increased local extracellular lipid concentrations, serve as ligands for the Toll-Like Receptor 4 (TLR4) complex and thereby activate the classical inflammatory response—which subsequently leads to macrophages infiltration in AT [72, 86]. During periods of a positive-energy balance, white AT expands to meet the need for extra triglyceride storage. This expansion is critically dependent on vascular supply [87]; for example, in the cases of increased adipocyte volume, oxygen is required to diffuse over longer distances before reaching adipocyte mitochondria. Furthermore, AT hypertrophy consequently creates areas of local microhypoxia, which activates and stabilizes the hypoxia-inducible factor 1 (HIF1)—which is a key hypoxia regulator [88, 89]. However, contrary to most other tissues, the hypoxic response of adipose tissue is insufficient to induce vascularization [32, 90], and instead, hypoxic AT increases the expression of profibrotic genes and leads to tissue fibrosis [91]. Indeed, HIF1 activates several genes involved in the regulation of various extracellular factors, such as collagen, in addition to components involved in extracellular matrix remodeling, which in turn leads to the global fibrotic response of this tissue [91]. It has been reported that fibrosis on AT plays an important role in the dysfunction of this tissue [92] and a connection has been shown to exist between alterations in the AT extracellular matrix, adipocyte survival, and inflammation [92]. This state of fibrosis results in the increased level of stress of expanding adipocytes, as well as necrosis, which in turn triggers an increased infiltration by immune cells (such as macrophages), which ultimately mediates higher levels of tissue inflammation. It has been hypothesized that HIF1-induced fibrosis could be the key initiating factor for monocyte infiltration and inflammation, with hypoxia being an early determinant which limits healthy AT expansion [91].

While these mechanisms can act independently, these inflammatory and metabolic pathways are nevertheless highly interconnected.

## 9. What Is the Role of Immune Cells in the Adipose Tissue of Obese Individuals?

Yes. In the case of lean AT, there are various anti-inflammatory immune cells—such as eosinophils, Tregs, and Th2 cells—which secrete anti-inflammatory molecules and contribute to the activation of macrophages type M2, contributing to the maintenance of insulin sensitivity [11, 32, 93].

In contrast, in the state of obesity, there are changes in the number and activity of various immune cells (e.g., the number of proinflammatory immune cells increases and the number of anti-inflammatory immune cells decreases), which plays a prominent role in the progression of insulin resistance, with the production of several proinflammatory mediators [11, 76]—several of which are components of the inflammatory pathways implicated in insulin resistance, such as TNF-*α* [94]. In this state, the dominant cells are MMe macrophages (resulting from the polarization of macrophages under the influence of the above-described stimuli), Th1 cells, CD8 T cells, and mast cells, which secrete Th1-type cytokines, leading to a proinflammatory milieu and insulin resistance. Furthermore, in this situation, neutrophils—which are early responders to inflammatory responses [95]—infiltrate the AT, where they secrete elastase and stimulate macrophage infiltration and proinflammatory cytokine secretion. It has been demonstrated that mice without neutrophil elastase have low levels of AT inflammation with improved insulin sensitivity, which suggests that these cells play a role in the regulation of AT inflammation in the early stages of obesity [95].

In obesity, mast cell-derived tryptase levels are also increased, such as the number of these cells in AT [96]. Furthermore, it has been demonstrated that mice that lack mast cells demonstrate reduced inflammatory responses and a high level of insulin sensitivity. IL-6 and IFN-*γ* are produced by these cells and they play a major role in contributing to the positive effects of mast cells on systemic energy homeostasis [11]. Eosinophils stimulate M2 macrophage polarization in AT by secreting Th2-type cytokines, such as IL-4 and IL-13, and are responsible for 90% of IL-4 expression in AT [93]. It has been demonstrated that eosinophil-deficient mice have increased inflammatory responses and insulin resistance, which suggests that eosinophils could act as anti-inflammatory immune cells in obesity [11, 93]. T cells are divided into various subpopulations, including CD4, CD8, and NKT cells. Most subtypes of these cells help in the regulation of AT inflammation in obesity. VAT has higher levels of total T cells in obesity [11]. CD4 T cells differentiate into various subtypes of CD4 T cells, such as Th1 and Th17 (which mediate proinflammatory responses), and Th2 and Treg cells (which contribute to anti-inflammatory responses) [97]. In obese AT, the number of Treg cells is decreased, which leads to high levels of AT inflammation and insulin resistance. The relative decrease in anti-inflammatory T-cell types in comparison to proinflammatory T cells could be associated with the increased infiltration of circulating monocytes to AT, and subsequent macrophage alteration in this tissue [11, 98]. Obese AT has high levels of CD8 T cells, which can have a critical role in the regulation of macrophage polarization and activation. Depletion of these cells leads to decreased levels of proinflammatory cytokines, such as IL-6 and TNF-*α*, with augmented insulin sensitivity. It has been demonstrated that CD8 T cells could have a potential role in the initiation of the inflammatory cascade, with some of these cells being predominant in early stages of obesity, preceding the infiltration of macrophages in AT [99]. Furthermore, certain NKT cells can identify certain lipid species, which are usually very prevalent in the case of obesity. However, the precise function of NKT cells in AT inflammation remains controversial [11, 100].

In addition, obese B cells also play a proinflammatory role, through the secretion of obesity-induced IgG. The loss of B cells leads to a diminution in the number of CD8 T cells in VAT, with an associated improvement in insulin sensitivity [101]. It is not well known which subtypes of B cells are involved in AT inflammation, neither of which mechanisms underlies the immune response of B cells in obese AT.

Nevertheless, many unanswered questions remain—such as which cells are involved in the initiation of AT inflammation, and how does AT inflammation modulate its mass.

## 10. Which Other Changes Occur in AT in Obesity?

The adipose tissue of patients with obesity also shows an increase in lipolysis and capillary rarefaction.

Obesity is also frequently associated with alteration in lipolysis [102, 103]—with an increase in basal rates of lipolysis—which could contribute to the development of insulin resistance, as well as an impaired responsiveness to stimulated lipolysis (by the catecholamines, in a fasting state) [103, 104]. In the postabsorptive state, there is an acute regulation of adipose tissue lipolysis, in order to maintain the supply of energy substrates and enable an efficient storage of excess fuels during post-meal digestion. Chronic exposure to extreme nutritional states, such as obesity or starvation, also leads to metabolic adaptations—which include changes in lipolysis. Decreased insulin suppression in adipocyte lipolysis could contribute to enhanced basal lipolysis in obesity [102]. Furthermore, it is widely accepted that obesity induces a rarefaction of capillaries of AT, which could limit the delivery of nutrients and contribute to both adipocyte dysfunction and insulin resistance [105]. In addition, obesity also leads to endothelial cell activation in AT (for example, by expressing higher levels of adhesion molecules), which additionally contributes to the infiltration of immune cells [106].

## 11. Does Metabolically Healthy Obesity Reality Exist? Is This a Benign Condition?

Metabolically healthy obesity (MHO) appears to be an intermediate stage of metabolic disorders between healthy and metabolically unhealthy obesity (MUO), although individuals can shift to the MUO group over the years.

It is well recognized that individuals in the same BMI category can have substantial heterogeneity of metabolic features. There are examples of individuals with long-standing obesity who can be considered to be otherwise healthy. This phenomenon has been described previously [2, 107], with such individuals being referred to as MHO. The classification of MHO currently only refers to metabolic or cardiovascular complications, without considering other nonmetabolic complications, such as cancer, dementia, and osteoarthritis [108, 109]. The prevalence of MHO varies depending on the population studied and on the definition used [109] and constitutes a unique subset of features, which reduce metabolic and cardiovascular risk.

The underlying mechanisms that could explain the existence of this phenotype are still being elucidated. There is a growing evidence suggesting that subclinical inflammation and macrophage phenotypes could be the underlying mechanisms, which determine whether an individual is MHO or not [110, 111]. In adulthood, this phenotype is associated with low markers of inflammation compared to patients with MUO, which supports the concept of a more favorable inflammatory state.

Furthermore, in order to explain the transition from normal AT to an AT that leads to metabolic abnormalities, it has been proposed that MHO has a different capacity for adaptation to excess energy in AT [109, 112]. As described above, when increased fat storage is required, fat tissue needs to increase its storage capacity, and thus an increase in the number or size of adipocytes occurs—which must be accompanied by increased vascularization [113]. Metabolic disease appears in individuals whose AT has difficulty in expanding in the healthiest way [114]. It has also previously been demonstrated that MHO exhibits mesenchymal cells in the stroma of AT, with a greater capacity to differentiate in both bone and fat tissue with a lower degree of senescence [115].

Several types of AT exist, and it is well known that visceral fat has a direct association with the metabolic consequences of obesity, whereas subcutaneous fat is not associated with metabolic disease [116], as described above. A possible hypothesis is that MHO accumulates fat mostly through subcutaneous fat expansion, and that only when this capacity is decreased, or is lost, does the organism have to resort to other types which are associated with metabolic diseases. Indeed, it seems that the decisive feature of MHO is the absence of visceral fat accumulation. Lifestyle factors also appear to play a key role in distinguishing whether an individual is MHO or not [117], which, in turn, influences, as an example, the polarization state of macrophages [109]. Adipose tissue dysfunction could therefore be the determinant of obesity-associated metabolic complications.

The literature differs regarding whether individuals with MHO are truly healthy or not [9]. However, those studies which had a longer period of follow-up concluded that obese individuals suffer from an increased risk of adverse long-term diseases—even without metabolic abnormalities—when compared with metabolically healthy normal weight (MHNW) individuals [117, 118]. This suggests that MHO is a state, which is not entirely benign. A recent meta-analysis [119] also demonstrates that obese individuals with no metabolic abnormalities have a higher risk of CVD and heart failure when compared with normal weight individuals without metabolic abnormalities. In addition, it has previously been found that nearly 42% of MHO patients transitioned to metabolically unhealthy phenotypes within 10 years when followed longitudinally [120].

Nevertheless, it remains unknown whether MHO has genetic predisposing factors, and also whether it ultimately succumbs to the metabolic syndrome.

## 12. Conclusion

Obesity is a complex disease, whose metabolic consequences have been studied widely. This disease is associated with increased AT, which represents a special connective tissue that contains adipocytes and several other types of cells, which is surrounded by capillary and innervation networks which function together as an integrated unit. Although lipid storage, thermal activity, and mechanical insulation are all classical functions of AT, this tissue is a multifunctional and metabolically highly active, immune, and endocrine organ, which directly modulates many processes, including energy balance and metabolism. Different types of adipocytes exist, with white adipocytes being the crucial ones for energy storage. Furthermore, it is possible to classify adipose tissue as being either subcutaneous adipose tissue or visceral adipose tissue, and it has been demonstrated that the latter—which is determined by measures such as increased waist circumference or waist to hip ratio, as well as elevated intra-abdominal fat area in cross-sectional abdominal imaging—is strongly associated with increased cardiometabolic risk. Obesity is a state, which is characterized by an increase in inflammatory state, although this degree of inflammation differs depending on the distribution of fat accumulation, being higher if stored at the VAT level. There is a growing evidence to suggest that these differences could be the underlying mechanisms, which determine whether an individual is MHO or MUO.

Although some of the above-mentioned processes are relatively well characterized, additional studies are required to clarify their physiological roles more precisely, as well as the contribution of each individual cellular type of AT in determining AT inflammation and subsequent insulin resistance. Understanding the endocrine function of AT and the changes that occur in this tissue in states of obesity could result in discovering more rational approaches for treating the metabolic consequences of excess AT.

## Data Availability

The data used in this article are in the articles whose references we use throughout the text.
